# Transforming Crisis Airway Management: Clinical Applications of a Distal Pharyngeal Airway Innovation in Two Cases

**DOI:** 10.7759/cureus.110544

**Published:** 2026-06-09

**Authors:** Connie L Lorette, Sharon Pearce, Kurt Vernon

**Affiliations:** 1 Doctor of Nursing Practice-Nurse Anesthesia (DNP-NA) Program, Bouvé College of Health Sciences, Northeastern University, Boston, USA; 2 Anesthesiology, Sharon Pearce Anesthesia, Speaking and Consulting, Garner, USA; 3 Gastroenterology, The GI Guy, Dunn, USA; 4 Gastroenterology, Harnett Health System-Dunn, Dunn, USA; 5 Gastroenterology, WakeMed Cary Hospital, Cary, USA

**Keywords:** airway techniques, difficult airway management, distal pharyngeal airway, procedural sedation and analgesia, visualization of airway in thyroidectomy

## Abstract

Airway management failure remains one of the most consequential adverse events in clinical practice, with the potential for severe hypoxemia, cardiac arrest, and death. While traditional airway adjuncts, such as oropharyngeal airways (OPAs) and nasopharyngeal airways (NPAs), serve as foundational rescue tools, their limitations, including the inability to access distal pharyngeal obstructions, risk of laryngospasm, and incompatibility with positive pressure ventilation, underscore the need for innovative solutions. The distal pharyngeal airway (DPA) is a relatively novel device designed to extend beyond the tongue base to the level of the epiglottis, addressing the most common anatomic site of upper airway obstruction.

We present two cases that demonstrate the lifesaving utility of the DPA in high-acuity airway emergencies. The first case describes a 47-year-old male with a BMI of 46, a history of obstructive sleep apnea and multiple comorbidities, who experienced rapid oxygen desaturation to 60% during procedural sedation for esophagogastroduodenoscopy (EGD). The jaw thrust maneuver proved ineffective; insertion of the DPA device through the endoscopic bite block aperture with bag-valve-mask ventilation restored oxygen saturation within 30 seconds, and the procedure was completed successfully. The second case involves a 69-year-old female who presented in acute respiratory distress following thyroid lobectomy, with a postoperative hematoma causing tracheal displacement and complete obliteration of anterior neck landmarks. Sequential failed attempts with two video laryngoscopes and a supraglottic airway resulted in oxygen saturation declining to 60%. DPA placement with two-person assisted mask ventilation successfully restored ventilation and oxygenation, bridging the patient to definitive airway management and subsequent video laryngoscopy-assisted intubation with a bougie.

These cases highlight the DPA as an effective rescue device in both procedural and emergency airway settings, particularly when conventional adjuncts fail. Early recognition of high-risk scenarios, systematic decision-making, team coordination, and access to innovative devices such as the DPA are essential to optimizing patient safety and outcomes across diverse clinical environments.

## Introduction

Basic airway management remains one of the most critical skills in clinical practice, presenting significant challenges across diverse healthcare environments, including operating rooms, emergency departments, intensive care units, and non-operating room anesthesia settings such as endoscopy suites [1]. Failure to establish or maintain a patent airway and provide adequate ventilation can lead to potentially catastrophic outcomes, including severe hypoxemia, cardiac arrest, and death.

Esophagogastroduodenoscopy (EGD) is a commonly performed endoscopic procedure with greater than 7.4 million cases performed yearly [2]. Sedation during EGD has been found to improve patient comfort and quality of examination [3]. Airway management during sedation for endoscopic procedures presents unique challenges, particularly in patients with severe obesity and multiple comorbidities [4]. EGD, while generally considered low risk, can become life-threatening when sedation leads to rapid airway compromise. Hypoxemia is the most common sedation-related adverse event during endoscopy [5]. Patients with obstructive sleep apnea, high BMI, and cardiovascular or metabolic conditions are at increased risk for hypoventilation and hypoxemia during such procedures [5-7].

Similarly, surgical procedures involving the neck and upper airway, especially thyroidectomy, pose substantial risk for postoperative hematoma development and subsequent airway obstruction [8]. Airway management becomes more challenging when compounded by patient comorbidities, atypical airway anatomy, and acute emergency situations. Understanding these challenges and developing systematic approaches to airway assessment and management are essential for preventing adverse events and optimizing patient safety across all healthcare environments where airway intervention may be required [9].

This article presents two clinical cases demonstrating the successful use of a distal pharyngeal airway device (DPA), the McMurray Enhanced Airway, in challenging airway scenarios. Health Insurance Portability and Accountability Act authorization was obtained by both patients.

## Case presentation

Sedation-related airway obstruction during EGD (Case 1)

A 47-year-old male patient with a BMI of 46 and an American Society of Anesthesiologists class III designation was admitted to a freestanding endoscopy clinic for elective EGD. The patient had extensive comorbidities, including obstructive sleep apnea, gastroesophageal reflux disease, diabetes mellitus, hypertension, and cardiac dysrhythmias. Cardiology clearance was previously obtained. Airway examination revealed a Mallampati score of III with no history of anesthetic complications.

**Figure 1:**
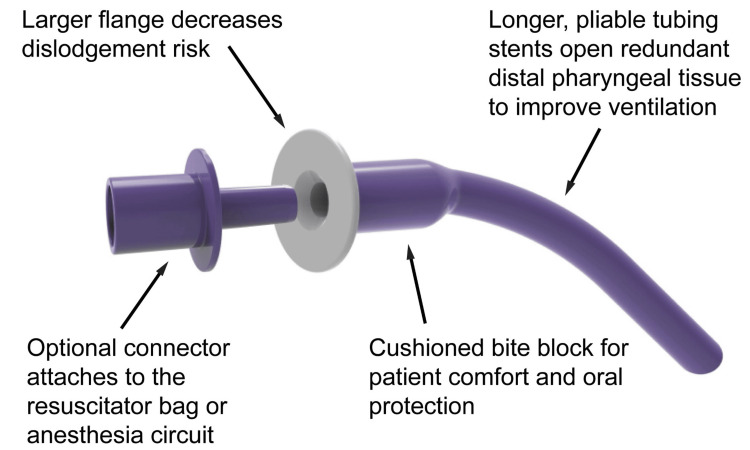
Design of the McMurray Enhanced Airway (MEA), a distal pharyngeal airway Permission was obtained for reproduction from McMurray Medical LLC. These images originate from internal educational materials, product documentation, and/or proprietary visual assets developed by McMurray Medical [10].

**Figure 2:**
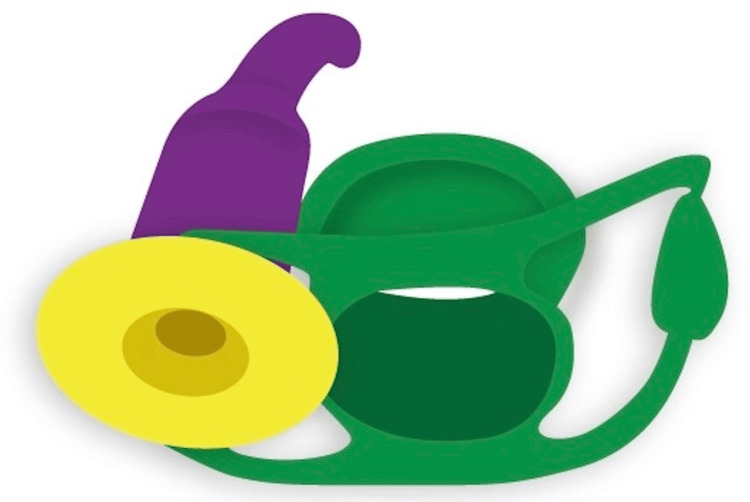
Distal pharyngeal airway positioned adjacent to a standard endoscopic bite block Permission was obtained for reproduction from McMurray Medical LLC. These images originate from internal educational materials, product documentation, and/or proprietary visual assets developed by McMurray Medical [10].

**Figure 3:**
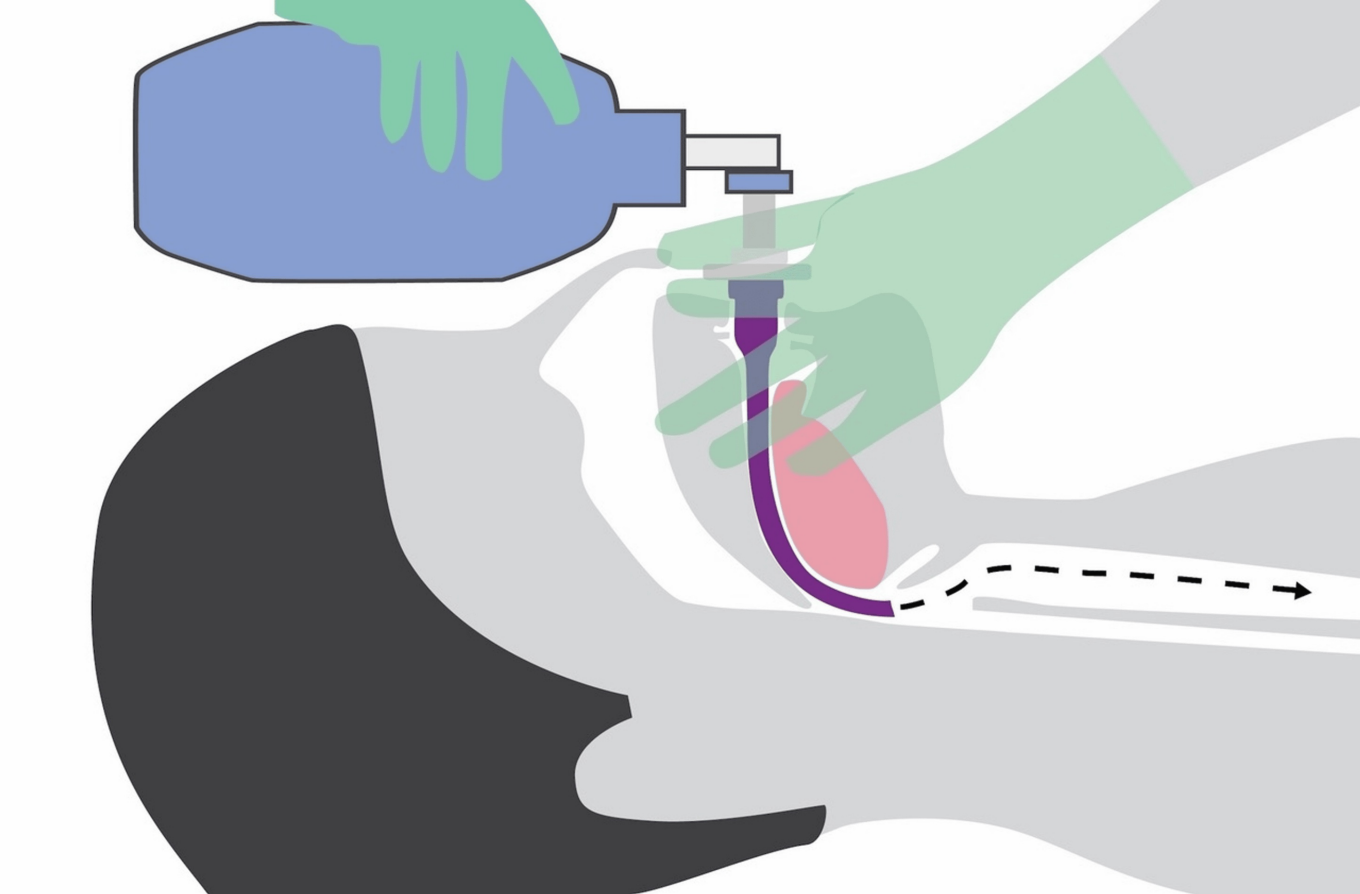
Intraoral ventilation using the distal pharyngeal airway with manual occlusion of the mouth and nares Permission was obtained for reproduction from McMurray Medical LLC. These images originate from internal educational materials, product documentation, and/or proprietary visual assets developed by McMurray Medical [10].

Following standard preparation with glycopyrrolate 0.2 mg IV, routine monitoring, and preoxygenation with O₂ 5 L/min via nasal cannula, the patient was positioned in left lateral decubitus with the head elevated 45 degrees. An endoscopic bite block was placed. Sedation was initiated with lidocaine 60 mg IV bolus and propofol 100 mg IV bolus, followed by an additional 100 mg of propofol IV bolus. The EGD scope was inserted with ease. Immediately following scope insertion, the patient’s oxygen saturation declined rapidly from 99% to 60%, prompting a jaw thrust maneuver. When this proved ineffective, the endoscope was removed, and a #4 DPA device, the McMurray Enhanced Airway (Figure 1), was inserted through the right aperture of the bite block (Figure 2). Positive pressure ventilation was initiated using a bag-valve-mask to the DPA device (Figure 3). End-tidal CO₂ was immediately detected, and oxygen saturation recovered within 30 seconds. The procedure was completed successfully, and the patient awoke without sequelae. 

Post-thyroidectomy hematoma airway emergency (Case 2)

A 69-year-old female patient presented to the emergency department shortly after being discharged following an uneventful thyroid lobectomy, complaining of incisional pain, neck swelling, and hoarseness. The patient had several comorbidities, including severe obesity, diabetes mellitus, hypertension, and tobacco use. A CT scan revealed an acute postoperative hematoma with tracheal displacement. Surgery and anesthesiology teams were consulted. The anesthesiology team arrived to find the patient in respiratory distress. The hematoma eliminated discernible anatomic landmarks on the anterior neck from the clavicle to the lower mandible. Emergent intubation was attempted under rapid sequence induction with propofol and succinylcholine. Multiple airway adjuncts were available, including two video laryngoscopes, supraglottic airways, fiberoptic bronchoscopy, and surgical airway equipment. Initial attempts with the two video laryngoscopes showed no visualization of vocal cords or epiglottis. A supraglottic airway was placed, but ventilation was unsuccessful. The patient’s oxygen saturation rapidly decreased to 60%.

While the emergency department physician was prepared to open the surgical site and possibly perform an emergency tracheostomy, a #4 DPA device was placed, and successful ventilation was established with two-person assisted mask ventilation. Oxygen saturation recovered, and ventilation was maintained for approximately three minutes until the surgeon arrived to evacuate the hematoma. The DPA device was then removed, and the patient was successfully intubated using video laryngoscopy with a bougie. The patient was transferred to the operating theater for further wound exploration and closure, remained intubated in the ICU, and was extubated without incident on post-operative day two, being discharged home on post-operative day three.

## Discussion

Traditional oropharyngeal airways (OPAs) and nasopharyngeal airways (NPAs) have long served as foundational basic airway adjuncts to improve ventilation and oxygenation by relieving upper airway obstruction. However, it is well documented that OPAs may trigger a gag reflex or laryngospasm and can push the tongue posteriorly, causing further obstruction, while NPAs carry a significant risk of epistaxis and aspiration [11,12]. When basic adjuncts fail, clinicians turn to advanced airway devices, such as video laryngoscopy, which has superior first-pass success rates over direct laryngoscopy [13], and supraglottic airways that can reduce severe hypoxemia during failed intubation [9]. However, a critical gap remains between current basic and advanced airway techniques, which is the need for an effective basic rescue device when traditional airways prove inadequate but advanced interventions are not immediately feasible or appropriate. 

The two cases presented illustrate how the DPA, a relatively new advancement in basic airway management, addresses this gap. In the first case, despite routine pre-oxygenation and appropriate sedation protocols, rapid desaturation occurred in a patient with significant comorbidities during procedural sedation. The DPA provided prompt resolution. In the second case, a post-thyroidectomy hematoma created an extremely challenging scenario where conventional advanced airway techniques failed. The DPA ultimately enabled successful ventilation and patient stabilization. 

The DPA's effectiveness stems from its unique design, as it extends beyond the tongue to open distal pharyngeal tissue near the epiglottis, which is the common site of obstruction that traditional OPAs and NPAs cannot reach. Moreover, unlike conventional airways, the DPA maintains compatibility with positive pressure ventilation and can be used even in the presence of an endoscopic bite block [10]. This bridges the critical gap between ineffective basic airways and failed or inappropriate advanced techniques. 

These experiences underscore essential principles for airway management: early recognition of high-risk scenarios, a systematic approach to assessment, and most critically, immediate availability of versatile rescue devices. Integration of innovative tools like the DPA into emergency airway protocols requires institutional support through appropriate training and clinical guidelines. As these cases demonstrate, the difference between successful rescue and catastrophic outcomes depends on having the right rescue options immediately available.

## Conclusions

The case reports demonstrate the DPA’s effectiveness as a rescue airway device in two critical scenarios when conventional methods failed: procedural sedation-related obstruction and post-thyroidectomy hematoma. The DPA’s unique design, providing distal pharyngeal access beyond traditional airways, enabled successful positive pressure ventilation when both basic and advanced techniques proved inadequate. Clinicians should consider incorporating DPAs into emergency airway protocols across all settings where airway intervention may be required, supported by appropriate training and institutional guidelines to enhance patient safety.
